# Superlubricity of Silicon-Based Ceramics Sliding against Hydrogenated Amorphous Carbon in Ultrahigh Vacuum: Mechanisms of Transfer Film Formation

**DOI:** 10.1021/acsami.3c16286

**Published:** 2024-01-31

**Authors:** Takuya Kuwahara, Yun Long, Aslihan Sayilan, Thomas Reichenbach, Jean Michel Martin, Maria-Isabel De Barros Bouchet, Michael Moseler, Gianpietro Moras

**Affiliations:** † Fraunhofer IWM, MikroTribologie Centrum μTC, Wöhlerstraße 11, 79108 Freiburg, Germany; ‡ Department of Mechanical Engineering, Osaka Metropolitan University, 3-3-138 Sugimoto, Sumiyoshi-ku, 558-8585 Osaka, Japan; § Laboratory of Tribology and System Dynamics, CNRS, UMR5513, University of Lyon, Ecole Centrale de Lyon, 69130 Ecully, France; ∥ Institute of Physics, University of Freiburg, Hermann-Herder-Straße 3, 79104 Freiburg, Germany; ⊥ Freiburg Materials Research Center, University of Freiburg, Stefan-Meier-Straße 21, 79104 Freiburg, Germany; # Cluster of Excellence livMatS, Freiburg Center for Interactive Materials and Bioinspired Technologies, University of Freiburg, Georges-Köhler-Allee 105, 79110 Freiburg, Germany

**Keywords:** tribology, superlubricity, diamond-like carbon, silicon nitride, quantum-chemical molecular dynamics, transfer film, dry friction

## Abstract

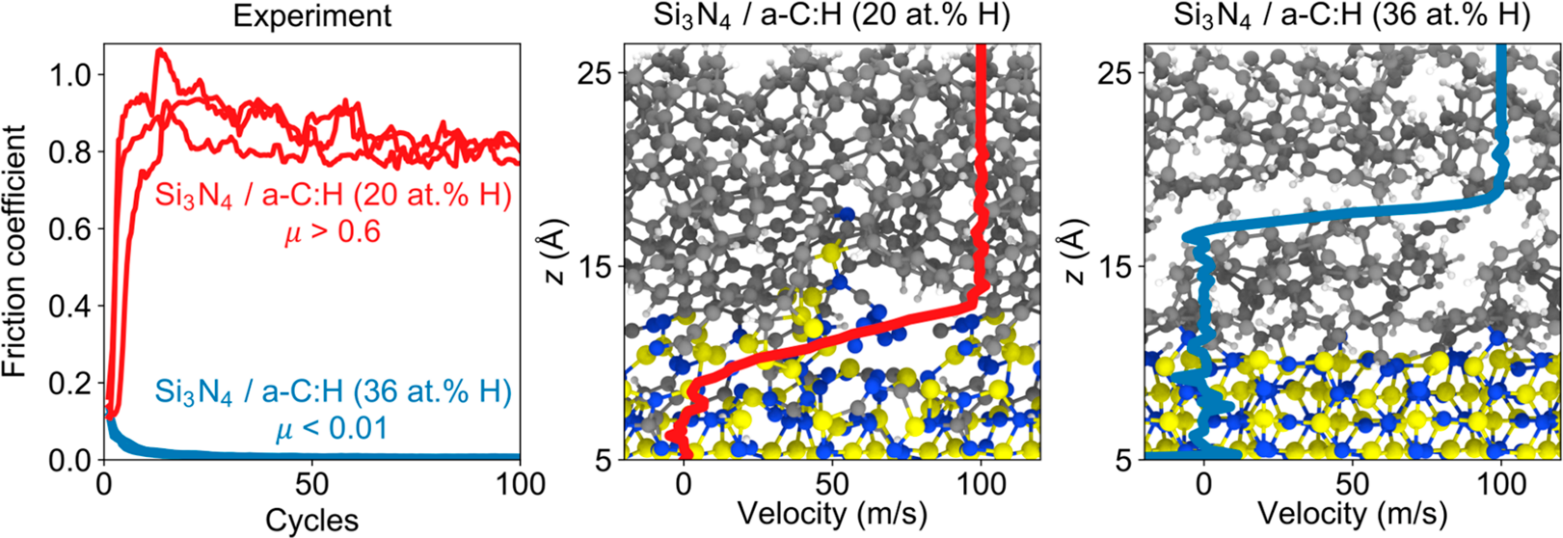

Tribological interfaces between silicon-based ceramics, such as Si_3_N_4_ or SiC, are characterized by high friction and wear in unlubricated conditions. A solution to this problem is to use them in combination with a hydrogenated amorphous carbon (a-C:H) countersurface from which a passivating carbon film is transferred onto the ceramic surface. However, the mechanisms underlying a stable film transfer process and the conditions that favor it remain elusive. Here, we present friction experiments in ultrahigh vacuum in which friction coefficients lower than 0.01 are achieved by sliding Si_3_N_4_ against a-C:H with 36 at. % hydrogen but not against a-C:H with 20 at. % hydrogen. Chemical surface analyses confirm that the superlubric interface forms via the transfer of a hydrocarbon nanofilm onto the Si_3_N_4_ surface. Quantum-mechanical simulations reveal that a stable passivating a-C:H film can only be transferred if, after initial cold welding of the tribological interface, the plastic shear deformation is localized within the a-C:H coating. This occurs if the yield shear stress for plastic flow of a-C:H is lower than that of the ceramic and of the shear strength of the a-C:H-ceramic interface, i.e., if the a-C:H hydrogen content ranges between ∼30 and ∼50 at. %. While the importance of a relatively high hydrogen content to achieve an efficient passivation of a-C:H surfaces in a vacuum is well-documented, this work reveals how the hydrogen content is also crucial for obtaining a stable a-C:H transfer film. These results can be extended to glass, SiC, and steel, supporting the generality of the proposed mechanism.

## Introduction

Silicon-based ceramics, such as silicon nitride (Si_3_N_4_) and silicon carbide (SiC), have prominent mechanical and thermal properties that make them materials of choice in many tribological applications.^1−3^ Under certain lubrication conditions, these materials have exceptional friction properties. For instance, superlubricity (i.e., friction coefficient μ lower than 0.01) was reported for self-mated Si_3_N_4_ contacts under lubrication with water,^4,5^ aqueous solutions,^6^ and glycerol.^7^ However, friction and wear can become very high if the contacts between these ceramic materials run dry.^8,9^ One of the most promising ways to tackle this problem is to induce the formation of protective, low-friction carbon tribofilms on metal and ceramic surfaces by means of tribochemical reactions of lubricants in boundary lubrication conditions.^7,10−14^ An alternative path that can lead to the formation of a stable passivating tribofilm on a silicon-based ceramic surface consists in replacing the ceramic countersurface with a solid lubricant^15^ that shows excellent lubricity in unlubricated contacts. For example, this strategy has been adopted in the past to achieve superlubricity of steel sliding against hydrogenated amorphous carbon in a vacuum.^16,17^

Hydrogenated amorphous carbon (a-C:H) belongs to the wider class of diamond-like amorphous carbon (DLC) materials^18^ and is an excellent solid lubricant that can exhibit superlubricity in a vacuum or dry environments.^19,20^ Superlubricity of a highly hydrogenated a-C:H (with a hydrogen content *C*_H_ ≈ 40 at. %) in ultrahigh vacuum (UHV) was first reported in the early 2000s.^19,20^ Superlubricity of a less hydrogenated a-C:H with *C*_H_ ≈ 34 at. % was observed only in H_2_ gas but not in UHV,^19^ indicating that in this case reactions of H_2_ molecules with a-C:H surfaces are necessary to passivate surface dangling bonds. The friction reduction is thus attributed to the surface passivation, which prevents the formation of strong covalent bonds across the sliding interface, and at the same time is characterized by the Pauli repulsion between passivating C–H groups.^21^ Without H_2_ gas, e.g., in an inert N_2_ atmosphere, a hydrogen-depleted a-C:H surface undergoes graphitization,^22^ and the formation of a graphitic lamellae nanolayer is also able to trigger a-C:H’s superlubricity due to its high degree of aromaticity.^7,23^

By exploiting the excellent properties of a-C:H coatings in unlubricated conditions, superlubricity was observed experimentally for Si_3_N_4_ sliding against a-C:H with *C*_H_ ≈ 39 at. % in a N_2_ atmosphere.^22^ Superlubricity was made possible by the formation of a ∼5 nm thick carbonaceous transfer film on the Si_3_N_4_ surface. The reconstruction of the sliding interface was triggered by the adhesion between the two materials and by the resulting shear-induced plastic deformation in narrow sheared regions within the a-C:H coating. The realization of a contact between two passivated carbonaceous surfaces led to a friction reduction of the heterogeneous tribopair under unlubricated conditions. Experiments on different types of ceramics sliding against a less hydrogenated a-C:H (*C*_H_ ≈ 8 at. %)^24^ showed high friction and wear and that their different adhesion strengths to a-C:H determine the transfer film formation on the ceramics and thus the frictional behavior. However, despite previous experimental efforts^25−27^ to understand tribological properties of the unlubricated Si_3_N_4_/a-C:H interface, the detailed mechanisms underlying the transfer film formation and under which conditions it occurs remain elusive.

In this study, we combine tribometry and surface analysis experiments with atomistic simulations not only to show that superlubricity of silicon-based ceramics sliding against a-C:H can be achieved in UHV and is enabled by the formation of a carbonaceous nanofilm on the ceramic surfaces but also to shed light on the hitherto unknown atomistic mechanisms that are responsible for the film transfer. We find that the transfer of a stable passivating a-C:H film onto the ceramic surface is only possible if, after initial cold welding between the a-C:H and the ceramic surfaces, the plastic shear deformation is localized within the a-C:H coating. In our analyses, this occurs for a-C:H hydrogen contents between ∼30 and ∼50 at. % because within this hydrogen content range the yield shear stress at which plastic shear flow occurs in a-C:H is lower than the yield shear stress of both the ceramic material and the ceramic-a-C:H interface. Our results provide mechanism-based criteria to design a-C:H coatings that offer a compromise between yield shear stress and strong covalent bonding to the ceramic counterpart, thus leading to the transfer of carbonaceous passivating tribofilms on ceramic surfaces and to superlubricity under unlubricated conditions.

## Results

### Experiments

**Figure 1:**
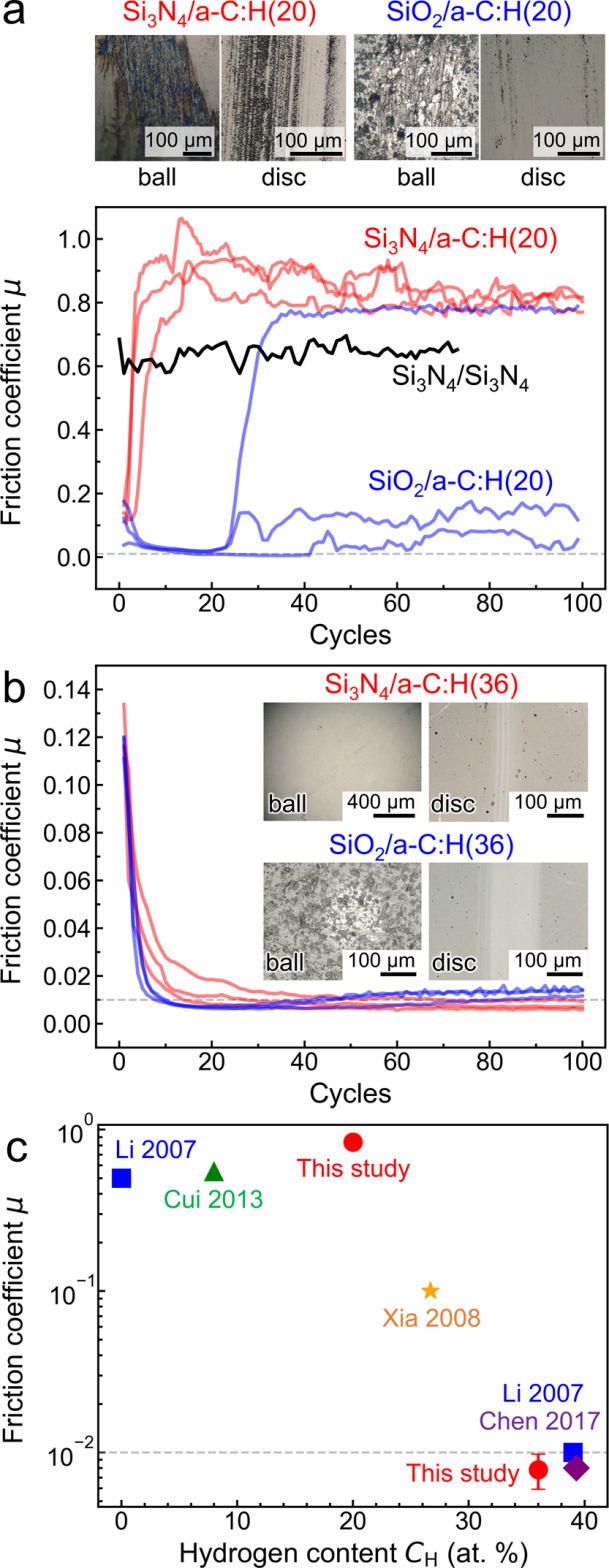
Reciprocating ball-on-flat friction tests of (a) Si_3_N_4_/a-C:H(20) and SiO_2_/a-C:H(20) tribological systems and (b) Si_3_N_4_/a-C:H(36) and SiO_2_/a-C:H(36). (c) Friction coefficients of the Si_3_N_4_/a-C:H tribological system measured in a vacuum and dry N_2_ as a function of the hydrogen content.^22,24−26^ Details of the measurements are provided in Table S1. In panel a, the black line is reported as a comparison and reproduced with permission from a previous reciprocating ball-on-flat test of a Si_3_N_4_/Si_3_N_4_ contact in UHV, where a similar sliding speed of 0.6 mm s^–1^ and normal load of 1.9 N were employed. Reproduced with permission from ref (9). Copyright 1991 Elsevier. The images in panels a and b are optical images of wear scars on ceramic balls and a-C:H-coated flats after the tests. In panels a and b, the tests are repeated three times to ensure reproducibility, and the horizontal lines represent the typical superlubricity threshold (μ ≈ 0.01).

Unlubricated, self-mated Si_3_N_4_ tribological contacts normally show high friction and wear in unlubricated conditions such as UHV (black line in Figure 1a, reproduced from ref (9)), and superlow friction has not been achieved in these conditions, to the best of our knowledge. This motivates us to explore an alternative pathway for achieving superlubricity with unlubricated Si_3_N_4_ and to investigate the friction properties of Si_3_N_4_ sliding against a-C:H surfaces. Reciprocating friction tests of an a-C:H-coated steel flat sliding against a Si_3_N_4_ and a glass (labeled as SiO_2_) ball are conducted in an UHV chamber (where the residual gas pressure is 5 × 10^–7^ Pa; further experimental details are provided in the Methods section). The SiO_2_ ball is simply used for comparison to understand whether the observed tribological behavior is only a result of the Si_3_N_4_ surface chemistry or whether it can also occur on the oxidized surface of a generic technological silicon-based ceramic. Two a-C:H coatings with different hydrogen contents, namely, *C*_H_ ≈ 20 and 36 at. %, are used in this study. These are hereafter denoted as a-C:H(20) and a-C:H(36), respectively. To remove adventitious carbon, the Si_3_N_4_ and SiO_2_ surfaces were sputtered with an Ar^+^ ion beam at 3 keV acceleration voltage for 6 min prior to the tests. In this way, the surface carbon content, as determined by X-ray photoelectron spectroscopy (XPS), decreases from ∼36 to ∼2 at. % on the Si_3_N_4_ ball and from ∼51 to ∼14 at. % on the SiO_2_ ball (Table 1). Impurities such as Al, Fe, and Ca are also found on Si_3_N_4_ balls, while Al, Na, K, and Ca are detected on SiO_2_ balls. These impurities amount to less than 5 at. % and originate from binders and glass-formers that are added during production. The full evolution of the chemical compositions of the Si_3_N_4_ and SiO_2_ surface as a function of sputtering time is shown in Figure S1 of the Supporting Information. Table 1 also shows that a large amount of oxygen (23 at. %) is present on the Si_3_N_4_ surface even after sputtering, suggesting that the Si_3_N_4_ surface is initially at least partially covered with a thin oxide layer. Because the oxide layer is soft, it can be easily removed by asperity collisions in running-in,^7^ leading to a contact between the Si_3_N_4_ and the a-C:H surfaces.

**Table 1:** Chemical Compositions (in at. %) Obtained from XPS Analyses on a Si_3_N_4_ and SiO_2_ Ball before Sputtering, before Sliding, and after Sliding against a-C:H(36)

Si_3_N_4_	C1s	O1s	Si2p	N1s	Al2p	Ca2p	Fe2p_3/2_
before sputtering	36	35	13	11	2	2	1
before sliding	2	23	32	35	2	1	4
after sliding	62	16	11	8	1	1	1

SiO_2_	C1s	O1s	Si2p	Al2p	K2p	Ca2p	Na1s
before sputtering	51	37	6	3	1	1	1
before sliding	14	56	18	6	3	1	2
after sliding	67	23	6.3	2	1	1	1

The results of the friction tests with Si_3_N_4_/a-C:H(20) and SiO_2_/a-C:H(20) are summarized in Figure 1a. Sliding of the Si_3_N_4_ ball against a-C:H(20) coatings yields high friction coefficients that are comparable to those obtained in the past with self-mated Si_3_N_4_ in UHV^9^ (black line in Figure 1a). For all three tests with Si_3_N_4_/a-C:H(20), the friction coefficients immediately increase to about 0.9–1.1, and scratches are observed on both flat and ball (Figure 1a). During the SiO_2_/a-C:H(20) tests, the friction coefficients for all three tests are lower than 0.1 for about 20–40 cycles. After running-in, in one of the three tests, the friction coefficient increases abruptly to about 0.8, and scratches are generated in the wear track (Figure 1a). The other two systems exhibit a slight increase of the friction coefficient, which eventually becomes unstable and ranges between 0.05 and 0.2. The low friction states during running-in can be attributed to an initial passivation of the SiO_2_ surface with silanol, siloxane groups, or residual carbonaceous surface layers. While asperity collisions cause depassivation and removal of soft glassy layers, occasional repassivation with siloxane groups could suppress cold welding of contacting asperities and thus prevent an increase of the friction coefficient to higher values. In contrast, stable superlubricity is observed when both Si_3_N_4_ and SiO_2_ balls slide against the a-C:H(36)-coated flats. In all three Si_3_N_4_/a-C:H(36) consecutive tests (Figure 1b), an initial friction coefficient of about 0.1 drops rapidly and reaches a steady-state value below 0.01. The lowest friction coefficient recorded in these three tests is 0.006. No visible wear is detected on the Si_3_N_4_ ball, and a wear scar (with a diameter of 82 μm, almost the same as a Hertzian contact diameter of 80 μm) is observed on the a-C:H-coated flat (inset of Figure 1b). In the three SiO_2_/a-C:H(36) tests, the friction coefficient drops to 0.006 within the first 20 cycles and increases slightly (μ ≈ 0.01) afterward (Figure 1b). After the sliding tests, scratches are observed on the SiO_2_ ball, and the wear scar has a diameter of 168 μm, which is slightly larger than the calculated Hertzian diameter of 160 μm.

The friction coefficients measured in this study for the Si_3_N_4_/a-C:H system are plotted as a function of *C*_*H*_ in Figure 1c together with those obtained in previous works^22,24−26^ on Si_3_N_4_/a-C:H tribological interfaces in a vacuum and dry N_2_ (details of the measurements are provided in Table S1). We also note that the UHV superlubricity is not limited to the specific Hertzian contact pressure and tribopairs presented in Figure 1. The Si_3_N_4_/a-C:H(36) system also exhibits superlubricity at higher normal loads of 5 and 8 N, corresponding to maximum Hertzian contact pressures of 788 and 921 MPa, respectively (Figure S2). Moreover, we observe superlubricity for other materials, namely, SiC and steel, sliding against a-C:H(36) in UHV (Figure S3). The result obtained for the steel/a-C:H(36) tribopair is consistent with previous UHV friction tests by Fontaine et al.^16^

**Figure 2:**
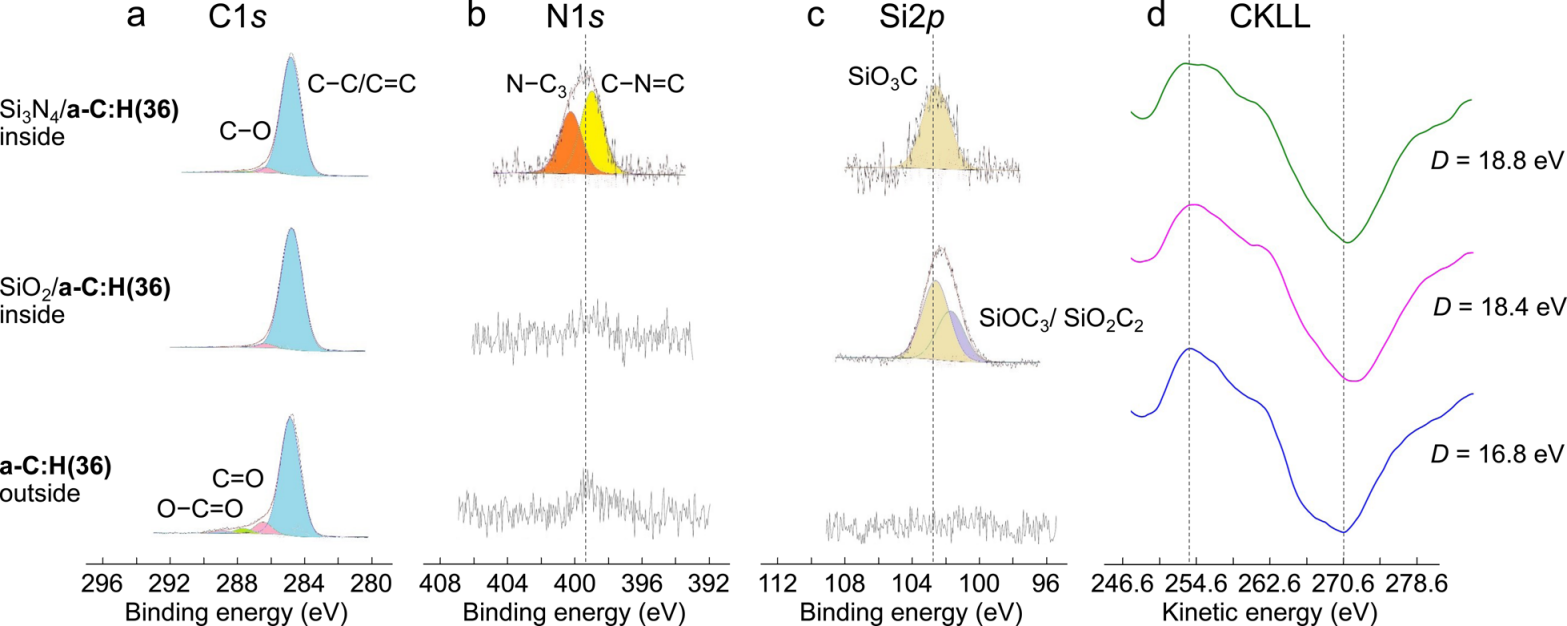
Surface chemical analyses of the a-C:H(36) coating inside and outside of the wear scar. High-resolution (a) XPS C1s, (b) N1s, and (c) Si2p spectra recorded inside the wear scar on the a-C:H(36)-coated steel flat after sliding against a Si_3_N_4_ ball (top) and a SiO_2_ ball (middle) and outside the wear scar (bottom). (d) First derivatives of XAES CKLL. The XPS binding energies are calibrated by referencing the central peak of C1s at 284.8 eV. Bold types in the left-hand side labels indicate the surfaces where XPS/XAES analyses are performed. The *D* parameters are defined as the kinetic energy difference between the maximum and minimum points in the first derivatives of the XAES CKLL spectra.

In order to obtain detailed information about the tribologically induced chemical and structural transformations at the sliding interface, XPS, Auger electron spectroscopy (XAES), and Raman spectroscopy analyses are performed for the Si_3_N_4_/a-C:H(36) and SiO_2_/a-C:H(36) systems. Owing to the large amount of wear, these chemical analyses were not performed on the high-friction systems with a-C:H(20). This does not affect the goals of this study, which focuses on the mechanisms of transfer film formation leading to superlubricity. Scanning secondary electron images are used to identify areas of interest and compare chemical compositions in different surface spots by energy dispersive X-ray spectroscopy (EDS). The XPS analyses for the a-C:H(36) coatings outside and inside the wear scar after sliding against the Si_3_N_4_ and SiO_2_ ball are summarized in Figure 2a–c. An analysis of the C 1s peaks does not reveal any remarkable difference between the areas inside and outside the wear scars, with a clear peak corresponding to C–C and C=C bonding visible in all three spectra (the spectra are calibrated by taking the C1s peak at 284.8 eV). However, a slight increase in the width of the C1s peak inside the a-C:H wear track is visible, which suggests an increase of the sp^2^ content. Because we have no accurate calibration of the C1s peak in this case, we do not try fitting the two contributions. A minor difference is observable for the peaks corresponding to C–O/C=O bonds, whose intensity is lower inside the wear scars than outside. Figure 2d also shows the first derivatives of the XAES CKLL spectra for the three cases. Here a difference between the *D* parameters outside and inside the wear scar is clearly visible (the *D* parameter is defined as the energy difference between the maximum and minimum points). Namely, *D* ≈ 16.8 eV outside the wear scar increases to 18.8 eV (Si_3_N_4_/a-C:H(36)) and 18.4 eV (SiO_2_/a-C:H(36)) inside the wear scars, possibly indicating an increase in sp^2^-hybridized carbon content after sliding,^28^ in agreement with the C1s peak width increase. Shoulder peaks around 262 eV corresponding to polymeric hydrocarbons are also visible for the three XAES CKLL spectra. Finally, the N1s spectrum shows a small C–N peak at 399.3 eV, corresponding to a relative content of about 1 at. % (not far from the XPS detection limit), detected on the a-C:H(36) surface after sliding against Si_3_N_4_ (Figure 2b). This peak can be attributed to pyridine or amine groups. Pyrrolic nitrogen is also recorded at 400.2 eV.^29^ As these N1s features are only visible inside the wear track of the a-C:H(36)-coated flat that was in contact with the Si_3_N_4_ ball, the small amount of nitrogen can then be attributed to a transfer from the Si_3_N_4_ ball to the a-C:H(36)-coated flat, and the analysis of the N1s peaks suggests the presence of N-containing carbon rings on the a-C:H(36)-coated flat.^7^ Similarly, small Si2p peaks appear only inside the wear scars on both a-C:H(36)-coated steel flats (Figure 2c). This also indicates that very small amounts of silicon on the ceramic surfaces are transferred onto the a-C:H(36) surfaces during sliding.

More important from the perspective of this study are the surface analyses of the Si_3_N_4_ and SiO_2_ balls after sliding. One of the most remarkable XPS results is the sharp increase in the carbon content after the tests on both ceramic balls: 2 → 62 at. % on the Si_3_N_4_ ball and 14 → 67 at. % on the SiO_2_ ball (Table 1). In contrast, the percentage of the other relevant surface chemical elements, i.e., Si, N, and O, decreases significantly, which is consistent with the growth of a C-rich tribomaterial on the ceramic surfaces. Because the experiments are performed in UHV and the only carbon source is a-C:H(36), the results strongly suggest that the carbonaceous layer that forms upon tribological load on both ceramics surfaces is a result of a film transfer from the a-C:H(36) surface. Taking into account the typical depth of analysis of XPS,^7^ the thickness of the carbonaceous transfer film can be estimated at less than 4 nm, which is in very good agreement with the scanning transmission electron microscopy results obtained by Chen et al.^22^ on analogous systems sliding in a dry N_2_ atmosphere.

**Figure 3:**
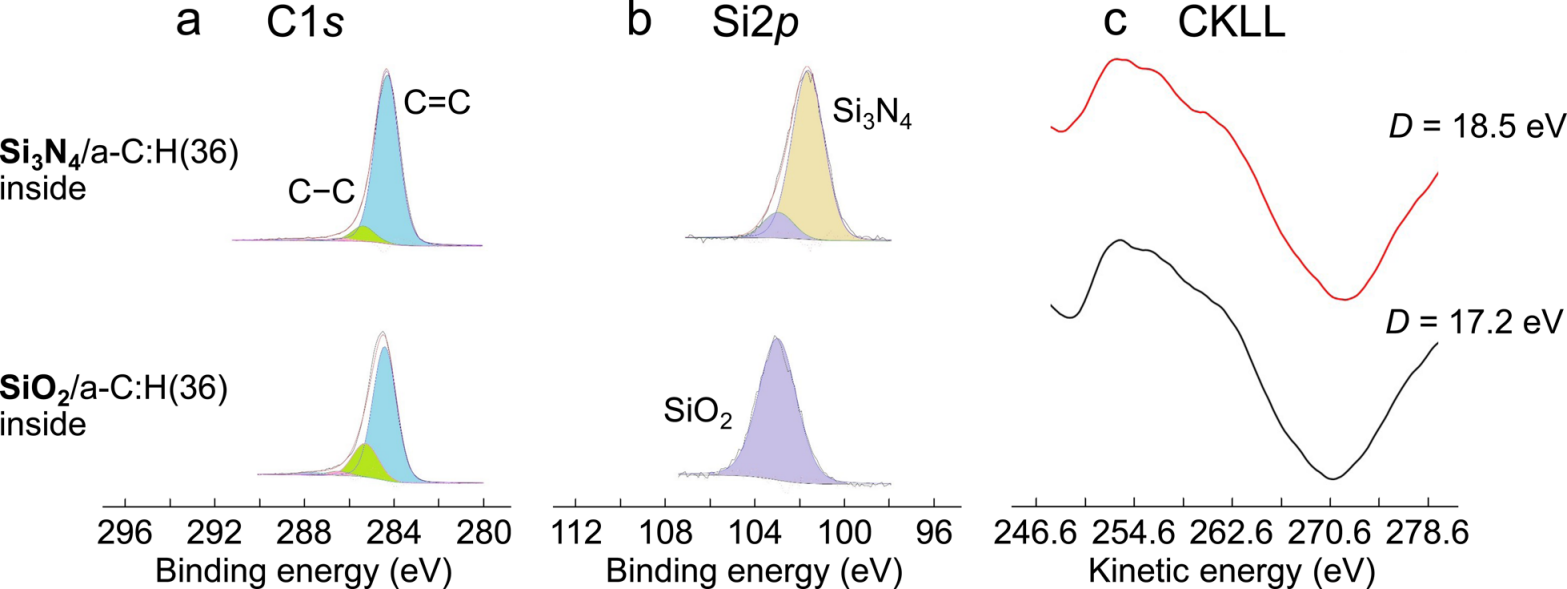
Surface chemical analyses of carbonaceous transfer films on ceramic balls. High-resolution (a) XPS C1s and (b) Si2p spectra recorded inside the wear scar on the Si_3_N_4_ (top) and the SiO_2_ ball (bottom). (c) First derivatives of the XAES CKLL spectra recorded inside the wear scar on the Si_3_N_4_ and SiO_2_ balls. For the XPS spectrum recorded on the Si_3_N_4_ ball, the binding energies are calibrated by referencing the Si_3_N_4_ peak at 101.7 eV. For the XPS spectrum recorded on the SiO_2_ ball, the binding energies are calibrated by referencing the SiO_2_ peak at 103.0 eV. Bold types in the left-hand side labels indicate the surfaces where XPS/XAES analyses are performed. The *D* parameters are defined as the kinetic energy difference between the maximum and minimum points in the first derivatives of the XAES CKLL spectra.

Large peaks in the XPS spectra, corresponding to C1s, are observed on both Si_3_N_4_ and SiO_2_ balls after the friction tests (Figures 3a and 3b, respectively). The C1s peaks recorded inside the wear scars on both Si_3_N_4_ and SiO_2_ balls are less symmetric than outside the wear scar on the a-C:H(36) surface (Figure 2a) and are fitted using two Gaussians corresponding to standard C–C and C=C energies for non-hydrogenated carbon (i.e., diamond and graphite) because those for hydrogenated carbon have not been well defined yet. Nevertheless, these XPS C1s spectra on both ceramic surfaces clearly show that a carbonaceous film is transferred on the ceramic surfaces during sliding and that the chemical structures of the transferred films are slightly different from those recorded inside and outside the wear scar on the a-C:H(36)-coated flat (Figure 2). The main C1s peaks on the ceramic balls are slightly shifted toward lower energies and have smaller contributions from C–O/C=O bonds compared with those recorded inside and outside the wear scar on the a-C:H(36)-coated flat. The larger width of the C1s peaks and their shifts with respect to the C1s peaks measured on the a-C:H(36) surfaces might also be caused by bonding of carbon to the silicon atoms of the underlying ceramic materials. Figure 3c shows the first derivatives of the XAES CKLL spectra recorded inside the wear scars on the Si_3_N_4_ and SiO_2_ balls. In this case, the *D* parameters are 18.5 and 17.2 eV for the Si_3_N_4_ and SiO_2_ balls, respectively, i.e., larger than the *D* parameter recorded outside the wear scar on the a-C:H flat (*D* = 16.8 eV, Figure 2d). In addition, shoulder peaks around 262 eV, attributed to the presence of polymeric chains −(CH_2_–CH_2_)_*n*_–,^23^ are less pronounced inside the wear scars on the ceramic balls than inside and outside the wear scars on the a-C:H(36)-coated flats. These spectroscopic analyses confirm the presence of a carbonaceous transfer film with a thickness of a few nanometers on the ceramic surfaces and suggest sp^3^-to-sp^2^ rehybridization and dehydrogenation of the transfer films.^28^ Supplementary Raman spectroscopy analyses of the SiO_2_ ball and a-C:H(36)-coated steel flat also confirm the presence of a carbonaceous transfer film on the SiO_2_ ball (Figure S4). The Raman spectra measured inside the wear scar on the SiO_2_ ball show the appearance of a clear D and G peak (at 1325 and 1596 cm^–1^, respectively) and a G-peak shift to a higher wavenumber compared with those measured on the a-C:H-coated flat. The spectra are similar to those published by Chen et al. for a Si_3_N_4_/a-C:H (with *C*_H_ ≈ 39 at. %) contact sliding in a dry N_2_ atmosphere.^22^ Finally, inside the wear scar on the Si_3_N_4_ surface, a C–N peak in the XPS N1s spectra is detected around 399.2 eV (Figure S5). This C–N peak is not detected outside the wear scar on the Si_3_N_4_ ball (Figure S5), thus confirming the presence of a tribologically induced carbonaceous transfer film on the Si_3_N_4_ ball (Figure 3a).

In summary, we observe stable UHV superlubricity of both Si_3_N_4_ and SiO_2_ sliding against a-C:H(36) but not against a-C:H(20). Chemical analyses of the surfaces indicate that superlubricity is related to the formation of a partially sp^2^-hybridized carbonaceous film with a few nanometers thickness on the ceramic balls during running-in. The transfer film formation gives rise to a sliding interface between two carbonaceous surfaces that are most likely passivated by hydrogen and aromatic structures,^22^ thus explaining the superlow friction coefficient in UHV.

### Simulations

Our experimental results suggest a possible correlation between the superlubricity of the Si_3_N_4_/a-C:H(36) system in UHV and the transfer of an a-C:H film onto the ceramic surface. The film transfer occurs for the Si_3_N_4_/a-C:H(36) system, which shows superlubricity, while for the Si_3_N_4_/a-C:H(20) system the surface chemistry was not analyzed because of the significant wear that accompanied the high friction coefficient (Figure 1). This high-friction state is compatible with the behavior reported in the literature for UHV sliding of self-mated Si_3_N_4_ surfaces^9^ and of ceramic surfaces against a-C:H coatings with a relatively low hydrogen content^24^ (*C*_H_ ≈ 8 at. %) (Figure 1c). This suggests that the Si_3_N_4_ surface was not protected by a passivated carbonaceous film. These considerations lead us to hypothesize the existence of a critical hydrogen content *C*_H_^*c*^ in the a-C:H coating above which a-C:H is transferred to the ceramic surface and the formation of a low-friction, passivated a-C:H/a-C:H sliding interface is enabled. The film transfer process could be determined by the competition between the yield shear stresses to generate shear flow of the two materials and the strength of the interface between them. More specifically, the yield shear stress of an a-C:H coating with a relatively high hydrogen content could be lower than that of the ceramic and of the cold-welded interface between the a-C:H coating and ceramic material. This could determine the formation of a “weak” shear interface within the amorphous carbon coating, ultimately leading to film transfer.

**Figure 4:**
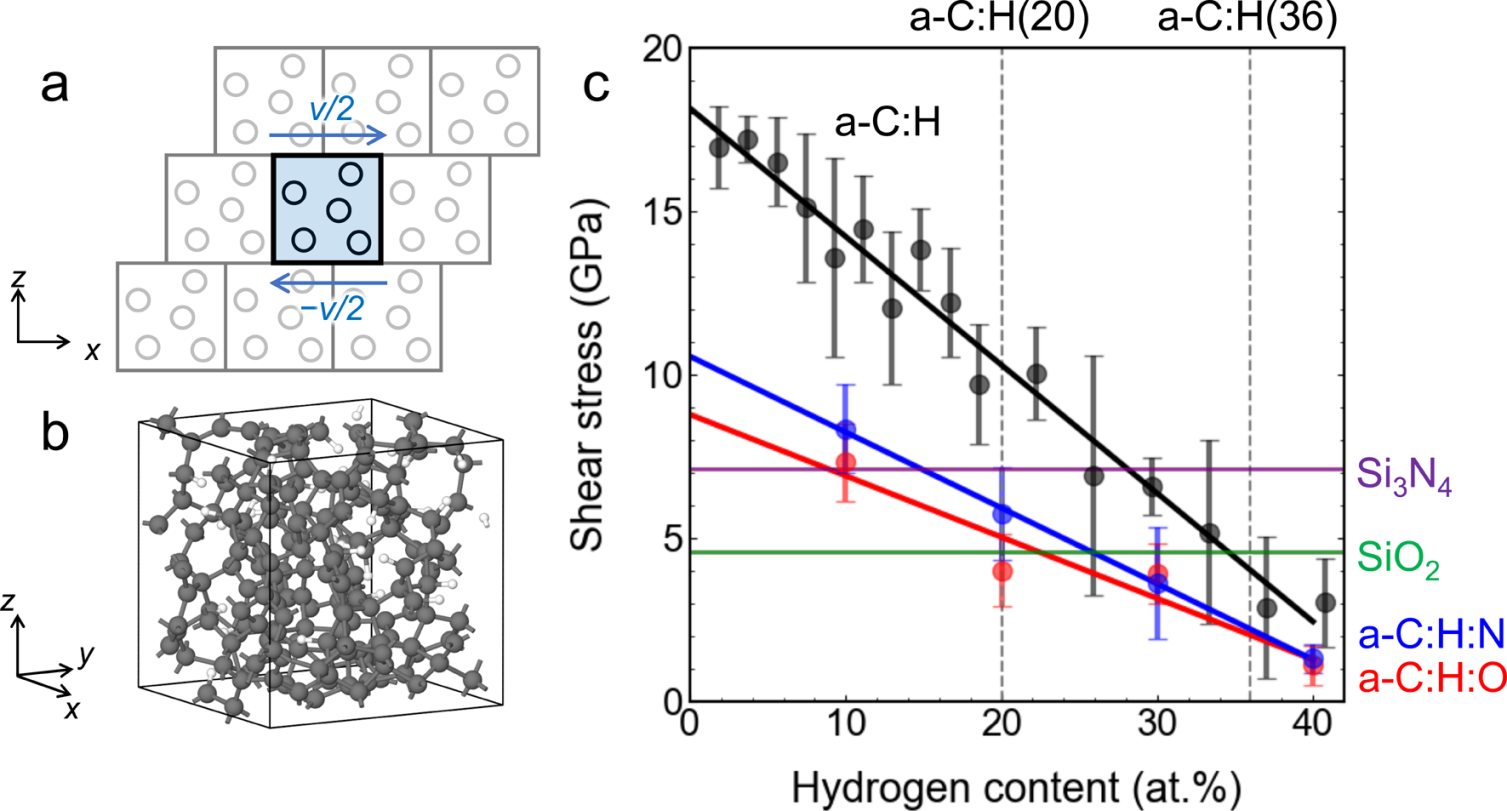
SCC-DFTB MD shearing simulations of bulk a-C:H samples with periodic Lees–Edwards boundary conditions (BCs).^31^ (a) A schematic illustration of Lees–Edwards BCs. (b) An example of the a-C:H bulk samples used here. Gray and white spheres represent carbon and hydrogen atoms, respectively. (c) Yield stress for plastic shear flow of a-C:H (black) as a function of the hydrogen content and its comparison with Si_3_N_4_ (purple) and SiO_2_ (green). The yield stress for plastic shear flow is calculated from 100 ps MD simulations by averaging the *zx* component of the stress tensor. A shear velocity of 100 m s^–1^ is applied along the *x* direction. An external pressure of 5 GPa is applied using a Berendsen barostat.^32^ The temperature is kept constant at 300 K using a Peters thermostat.^33^ Five trajectories are generated with different initial amorphous structures to obtain the shear stress at each point. In each trajectory, the amorphous solid undergoes a homogeneous shear deformation, and no localized low-shear interfaces form within the simulation time. The error bars represent standard errors of the means. Standard deviations of the means for SiO_2_ and Si_3_N_4_ are 0.12 and 0.30 GPa, respectively. For a-C:H:O (red) and a-C:H:N (blue), 10 at. % of the O and N are added into a-C:H, respectively. The vertical dashed lines indicate the hydrogen contents of the a-C:H(20) and a-C:H(36) coatings used in the experiments.

As a first step to substantiate these hypotheses, we employ quantum-mechanical, self-consistent-charge density-functional tight-binding^30^ molecular dynamics (SCC-DFTB MD) simulations to estimate the yield shear stresses at which plastic shear flow occurs in a-C:H, amorphous SiO_2_, and crystalline β-Si_3_N_4_ representative volumes upon uniform shear deformation. A homogeneous shear flow is generated using the Lees–Edwards boundary conditions (BCs).^31^ A schematic representation of the Lees–Edwards BCs and an example of the initial atomic configuration are depicted in Figures 4a and 4b, respectively. More technical details are available in the Methods section. This simulation mimics a contact between two surfaces, i.e., an asperity collision that occurs during running-in, upon which the two surfaces undergo cold welding and the materials flow plastically under the external load (e.g., during the film transfer process for the first 20 cycles in Figure 1b). A comparison of the yield shear stress of the a-C:H coatings with those of SiO_2_ and Si_3_N_4_ allows us to qualitatively predict the location of the shear plane as a function of *C*_H_. Plastic flow is more likely to localize in the material with lower yield shear stress, thus determining the film transfer process.

Figure 4c shows that the yield stress for plastic shear flow decreases approximately linearly as the a-C:H’s hydrogen content *C*_H_ increases (from ≈18 GPa at *C*_H_ = 0 at. % to ≈3 GPa at *C*_H_ = 41 at. %). Compatibly with our hypothesis, the yield shear stress for a-C:H becomes smaller than the shear stress of Si_3_N_4_ (τ = 7.1 GPa, purple line) at a critical value *C*_H_^*c*^ ≈ 28 at. %. For SiO_2_ (τ = 4.6 GPa, green line) the critical hydrogen content is *C*_H_^*c*^ ≈ 35 at. %. These critical values become even smaller if contaminants are present on the a-C:H surface, as suggested by our XPS analyses. For instance, the shear stress of a-C:H after incorporation of 10 at. % oxygen or nitrogen is shown by red and blue lines in Figure 4c, respectively. The dependence of the shear stress on the hydrogen content is still linear but *C*_H_^*c*^ decreases to *C*_H_^*c*^ ≈ 9–15 at. % for Si_3_N_4_ and *C*_H_^*c*^ ≈ 22–26 at. % for SiO_2_. These results confirm the existence of a critical a-C:H’s hydrogen content *C*_H_^*c*^ above which the yield stress for plastic shear flow of the a-C:H coating becomes lower than the analogous yield shear stress for the Si_3_N_4_ or SiO_2_ counterbody (we note that SiO_2_ here could represent a glass surface as well as a surface oxide film on the Si_3_N_4_ ball). Moreover, the simulations deliver threshold *C*_H_^*c*^ values that are broadly located between the hydrogen contents of the a-C:H coatings used in the experiments, i.e., 20 ≲ *C*_H_^*c*^ ≲ 36 at. %.

These results confirm that, in principle, after an initial cold welding between the a-C:H surface and the ceramic surface, the formation of a “weak” shear band within the a-C:H coating, within which the plastic shear deformation is accommodated, is possible if *C*_H_ > *C*_H_^*c*^. However, this can only happen if the shear stress that would cause sliding at the a-C:H/ceramic interface is larger than the yield stress for plastic shear flow in a-C:H. If the two surfaces are sufficiently passivated and the surface density of covalent bonds between the a-C:H and the ceramic surface is low or zero, then the shear deformation is likely to be accommodated at the initial interface, thus preventing the transfer of an a-C:H film onto the Si_3_N_4_ or SiO_2_ surface.

**Figure 5:**
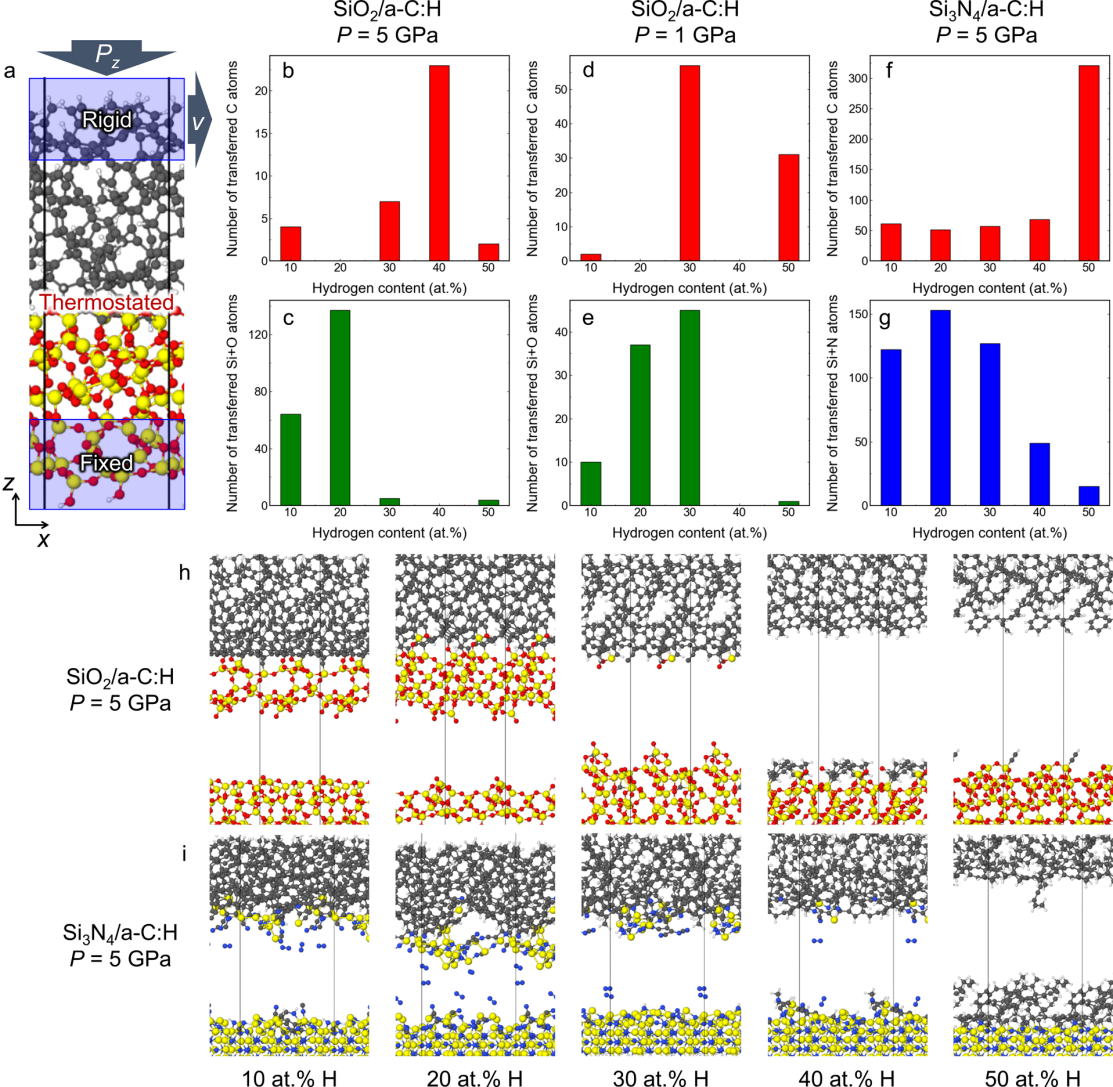
SCC-DFTB MD sliding and detachment simulations of SiO_2_/a-C:H and Si_3_N_4_/a-C:H interfaces. (a) Example of the simulation model for the interface between two materials using a pressure-coupling algorithm.^35^ Number of (b) C atoms that are transferred from a-C:H onto SiO_2_ and of (c) Si + O atoms transferred from SiO_2_ onto a-C:H at SiO_2_/a-C:H contacts with *P*_*z*_ = 5 GPa. Number of (d) C atoms transferred from a-C:H to SiO_2_ and of (e) Si + O atoms transferred from SiO_2_ onto a-C:H at SiO_2_/a-C:H contacts and *P*_*z*_ = 1 GPa. Number of (f) C atoms transferred from a-C:H onto Si_3_N_4_ and (g) Si + N atoms transferred from Si_3_N_4_ onto a-C:H at Si_3_N_4_/a-C:H contacts and *P*_*z*_ = 5 GPa. Examples of final atomic configurations after detachment for (h) a-C:H/SiO_2_ and (i) a-C:H/Si_3_N_4_ contacts at *P*_*z*_ = 5 GPa with varying hydrogen contents. Gray, red, yellow, blue, and white spheres represent C, O, Si, N, and H atoms, respectively.

To investigate this crucial aspect, we consider explicitly the SiO_2_/a-C:H interface (which models both the experimental interfaces between a-C:H and SiO_2_ and between a-C:H and oxidized silicon nitride) and the Si_3_N_4_/a-C:H interface. We perform SCC-DFTB MD sliding simulations of these interfaces with varying hydrogen contents (10 ≤ *C*_H_ ≤ 50 at. %) followed by the separation of the two material systems during sliding, which mimics the contact and detachment of two surface asperities during sliding. Owing to the limited time scale accessible by these simulations, it is impossible to describe the full film transfer process, which possibly relies on repeated contacts between surface asperities during running-in. Therefore, the goal of these simulations is to determine whether the probability of transferring carbon atoms from the a-C:H coating to the ceramic surface depends on the hydrogen content *C*_H_. An example of the simulation system for the interface between the two materials is shown in Figure 5a (details in the Methods section). For each tribological interface, five independent MD trajectories with different initial amorphous configurations are generated. The a-C:H samples used for the simulations with SiO_2_ and Si_3_N_4_ are different because the unit cell dimensions along the *x* and *y* directions are different. Thus, in total, ten a-C:H and five a-SiO_2_ samples are prepared. After sliding for 0.5 ns at a constant contact pressure *P*_*z*_ of 5 GPa, the upper rigid layer of the a-C:H slab is retracted from the contact by simultaneously removing the contact pressure and instead adding a vertical velocity of 20 m s^–1^ while keeping the sliding velocity constant. The choice of the local contact pressure *P*_*z*_ of 5 GPa is reasonable for stiff contacts between a-C:H and Si_3_N_4_^7,34^ independently of the presence of a thin surface oxide film. However, such contact pressure values are less probable for contacts between a-C:H and SiO_2_ owing to the lower elastic modules of SiO_2_. Thus, sliding simulations of the a-SiO_2_/a-C:H interfaces are performed at *P*_*z*_ = 1 and 5 GPa.

After detachment of the two surfaces, the number of carbon atoms transferred onto the ceramic surfaces and the number of silicon, oxygen, and nitrogen atoms transferred onto the a-C:H surfaces are counted. The results are summarized in Figure 5b–g for all sliding interfaces and contact pressures. The plots show the total sum over five simulations of transferred C atoms, Si + O atoms, and Si + N atoms. Representative configurations of the a-SiO_2_/a-C:H and Si_3_N_4_/a-C:H systems for different hydrogen contents after sliding with *P*_*z*_ = 5 GPa and detachment are depicted in Figures 5h and 5i, respectively. The material transfer process shows a dependency on *C*_H_ and rather large fluctuations in the number of transferred atoms associated with the particular chemical structure of the individual a-C:H systems. For a-SiO_2_/a-C:H contacts at *P*_*z*_ = 5 GPa, we observe that for *C*_H_ < 30 at. %, the transfer of C atoms to the a-SiO_2_ surface is significantly less probable than the transfer of Si and O atoms onto the a-C:H surface (Figures 5b and 5c). 1 nm thick a-SiO_2_ layers are transferred onto the a-C:H surfaces at *C*_H_ = 10 and 20 at. % (Figure 5h). The transfer of small amounts of C atoms for *C*_H_ < 30 at. % is ascribed to chemical mixing of the cold-welded system under shear (*C*_H_ = 30 at. %, Figure 5h). At *C*_H_ = 40 at. %, there is a maximum in the number of transferred C atoms, which decreases again at *C*_H_ = 50 at. %, most probably because of the hydrogen passivation of the a-C:H surfaces and the resulting weak interface between the a-SiO_2_ and a-C:H surface. For *P*_*z*_ = 1 GPa, the transfer of C atoms is observed at *C*_H_ = 30 and 50 at. %, but not at *C*_H_ = 40 at. %, probably because of the stochastic nature of the interfacial dynamics. No significant effects of the contact pressure are observed in our simulations.

The analysis of the Si_3_N_4_/a-C:H interfaces shows that at *C*_H_ ≤ 40 at. %, mutual transfer of atoms, i.e., carbon onto the Si_3_N_4_ surface (Figure 5f) and silicon/nitrogen onto a-C:H (Figure 5g), occurs as a result of amorphization of the Si_3_N_4_ surface and chemical mixing under shear. The observed transfer of nitrogen onto the a-C:H surface is in agreement with the XPS N1s spectra (Figure 3b). Representative snapshots after detachment at *C*_H_ ≤ 30 at. % (Figure 5i) show the transfer of amorphous silicon nitride layers onto a-C:H surfaces in addition to formation of N_2_ molecules. At *C*_H_ = 40 and 50 at. %, this mutual transfer of atoms is inhibited, and an a-C:H-derived transfer film forms on the Si_3_N_4_ surface (rightmost panel in Figure 5i).

**Figure 6:**
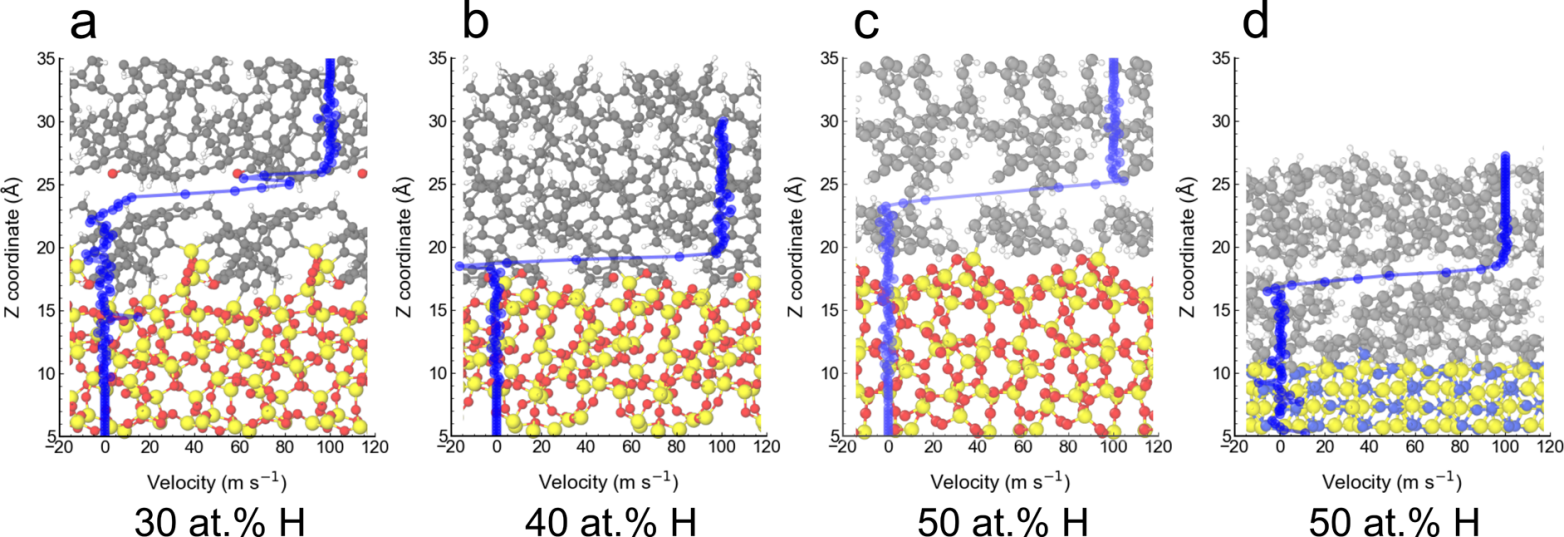
Atomic configurations and velocity profiles along the sliding direction for SiO_2_/a-C:H and Si_3_N_4_/a-C:H for different shear simulations in which the transfer of C atoms from a-C:H to the ceramic surface occurs before detachment: (a) SiO_2_/a-C:H, *P*_*z*_ = 1 GPa; (b) SiO_2_/a-C:H, *P*_*z*_ = 5 GPa; (c) SiO_2_/a-C:H, *P*_*z*_ = 1 GPa; and (d) Si_3_N_4_/a-C:H, *P*_*z*_ = 5 GPa. The snapshots represent atomic configurations at *t* = 0.5 ns just before the detachment. The velocity profiles are averaged over the last 0.1 ns before detachment. The configurations in panels b and d correspond to those in the fourth panel of Figure 5h and the rightmost panel of Figure 5i, respectively. The corresponding configurations to panels a and c are presented in Figure S7a,b, respectively.

The transfer of carbon atoms from a-C:H onto Si_3_N_4_ and SiO_2_ surfaces upon separation of the two sliding counterparts correlates well with the formation of weak shear interfaces inside a-C:H under normal load and sliding (Figure 6). Velocity profiles along the sliding direction are calculated for all three trajectories exhibiting a-C:H transfer in a-C:H/SiO_2_ contacts. For the three trajectories (Figure 6a–c), the velocity gradients are steep and localized in a-C:H, indicating the shear-induced formation of a low-friction interface inside the a-C:H even without detachment. This process begins with cold welding between the a-C:H and SiO_2_ surfaces, which is followed by plastic shear deformation at the interface. This leads to rearrangement of atoms near the sliding interface and to the self-formation of the easy-shear plane. The migration of the shear plane into the a-C:H coating can occur when a-C:H’s yield shear stress is lower than the yield shear stress of a-SiO_2_ and of the shear stress that causes sliding at the original interface (as shown in Figure 4). The time evolution of the shear stress τ for the corresponding trajectories (Figure S6) shows that τ is initially high (larger than a few GPa and close to yield shear stress of H-rich a-C:H) during the cold-welding phase but drops suddenly as low-friction interfaces form. The self-forming shear planes are identical with the interfaces after detachment, and similar running-in processes are observed for all three trajectories of the SiO_2_/a-C:H interface (final configurations at *t* = 0.5 ns in Figure 6a–c) and also for Si_3_N_4_ (Figure 6d). The atomic configurations after 0.5 ns of sliding in Figure 6 correspond to those after detachment shown in Figures 5 and S7 (see the one-to-one correspondence in the caption for Figure 6). These examples show a clear correlation between the localized shear plane in a-C:H under a normal load and film transfer process.

At least for SiO_2_ or oxidized Si_3_N_4_ surfaces the probability of formation of a carbon transfer film seems to be highest for 30 ≤ *C*_H_ ≤ 40 at. %, depending on the normal pressure. In general, for *C*_H_ < 30 at. %, the interface between a-C:H and SiO_2_ is characterized by a relatively high density of covalent bonds, and the velocity gradient tends to be accommodated inside the SiO_2_ owing to a-C:H’s higher yield stress for plastic shear flow. In contrast, at *C*_H_ > 40 at. %, hydrogen passivation of the a-C:H surface can inhibit cold welding between the a-C:H and the ceramic surfaces. In such cases, the shear plane is localized at the interface between the a-C:H and the ceramic, which prevents any transfer of material form one surface to the other.

## Discussion

Our reciprocating friction tests performed in UHV with Si_3_N_4_ and SiO_2_ balls sliding against a-C:H-coated steel flats show extremely different friction behaviors depending on the hydrogen content *C*_H_ of the a-C:H coatings. Si_3_N_4_/a-C:H(20) tribological systems show steady-state friction coefficients of about 0.8, which are compatible with cold welding of the sliding interface^9,24^ and significant wear. After initial depassivation, cold welding of the SiO_2_/a-C:H(20) interface is probably mitigated by self-passivation of the SiO_2_ surface with hydroxyl or siloxane groups, leading to an unstable friction coefficient of about 0.1 in two out of three measurements. Conversely, stable friction coefficients of about 0.01 or lower are consistently measured when the two ceramic materials slide against a-C:H(36) surfaces. In these cases, surface analyses reveal the presence of a carbon-rich tribofilm on the Si_3_N_4_ and SiO_2_ balls after sliding. This suggests that the superlubric sliding interfaces can be attributed to two passivated a-C:H surfaces sliding against each other, in agreement with previous studies reporting superlow friction coefficients for self-mated a-C:H tribological interfaces in UHV.^23^ At first sight, these results could be ascribed solely to the number of hydrogen-passivated carbon dangling bonds, which should increase with increasing *C*_H_. However, it should be noted that the passivation of a-C:H surfaces can be effectively achieved also at low *C*_H_, for instance by localized aromatization.^23^ Both passivation mechanisms require plastic rearrangement of a-C:H during running-in.^23^ Therefore, it is crucial to investigate the effect of *C*_H_ on the a-C:H plastic shear deformation and on the film transfer process.

Quantum-mechanical simulations provide insights into the atomic-scale mechanisms underlying the formation of a carbonaceous transfer film on silicon-based ceramic surfaces. The main outcome of the simulations is that the probability of a carbon-film transfer from the a-C:H coating to the ceramic countersurface is determined by the location of the shear interface. This in turn mainly depends on the relative yield stresses for plastic shear flow of (i) the a-C:H material, (ii) the ceramic topmost layer, and (iii) the interface between the two. In this context, the amount of hydrogen *C*_H_ in the a-C:H coating is crucial for the film-transfer process because it is one of the main factors determining the yield shear stress of both the a-C:H material and its interface to the ceramic counterbody.

The simulations reveal that for a-C:H coatings with a hydrogen content *C*_H_ lower than about 30 at. % the a-C:H surface is not readily passivated after a wear event, leading to cold welding of the a-C:H and the ceramic surface. In this case, the plastic shear region, in which the velocity gradients are accommodated, migrates to a region underneath the ceramic surface, as both Si_3_N_4_ and SiO_2_ oppose a lower resistance to plastic shear flow than the a-C:H coating and the ceramic/a-C:H interface. This causes the formation of a ceramic/ceramic sliding interface in UHV, which is characterized by high values of friction and wear, as shown by our experiments on a-C:H(20) and by other studies,^8,9,24^ all reporting friction coefficients of about 0.8.

An increase of *C*_H_ to values between about 30 and 50 at. % causes the yield shear stress of the a-C:H coating to decrease to values that are now lower than the yield shear stress of the ceramic (Figure 4) and of the cold-welded interface. As a result, a plastic shear interface forms within the a-C:H coating. The resulting shear-induced rearrangement of the atomic structure of a-C:H can lead to the formation of a superlubric interface between a-C:H surfaces that are passivated by H atoms and aromatic carbon structures (see Figure 6 and ref (23)). A further increase of *C*_H_ to values higher than about 50 at. % further decreases the a-C:H’s yield stress for shear flow but also significantly weakens the interface between the carbon coating and the ceramic owing to an effective hydrogen passivation of the carbon dangling bonds on the a-C:H surface. In this situation the most probable position of the sliding interface is between the a-C:H coating and the Si_3_N_4_ or SiO_2_ surface, and the carbon film transfer is thus suppressed, as indicated by a drop in the number of transferred carbon atoms at 40 < *C*_H_ < 50 at. % in Figure 5b. The higher density of dangling bonds on the Si_3_N_4_ surface, i.e. its higher reactivity, could allow the range of *C*_H_ where a film transfer occurs to be extended to higher hydrogen contents *C*_H_ compared with the SiO_2_ surface (as shown in Figure 5f). We note that fluctuations in the number of transferred C atoms observed in the simulations are caused by the nonequilibrium interfacial dynamics that depends on stochastic bond-formation and bond-breaking events. Thus, the film transfer process is not solely determined by the a-C:H’s hydrogen content. For example, the transfer film for *C*_H_ = 30 at. % (Figure 6a) is thicker than the film observed for *C*_H_ = 50 at. % (Figure 6c). Each a-C:H representative volume can intrinsically possess a nonuniform distribution of hydrogen along the direction in which the normal pressure is applied, i.e., a weak shear plane. The chemical structures near the sheared region even evolve during shear, which could result in the formation of a weak shear plane in a-C:H and thus a low-friction interface between a-C:H layers (Figure S8).

Overall, these results indicate that to maximize the likelihood of carbon film transfer, *C*_H_ must be high enough to ensure that the yield shear stress of a-C:H is lower than that of the ceramic material. At the same time, *C*_H_ should be low enough to ensure a strong bonding between the a-C:H and the ceramic surfaces. Experiments by Cui et al.^24^ on sliding contacts between Si_3_N_4_ balls and a-C:H-coated surfaces with *C*_H_ ≈ 8 at. % yielded friction coefficients of about 0.6, which are consistent with our simulations. The results of our simulations suggest an optimal *C*_H_ between 30 and 40 at. %, in agreement with our experimental results that show superlubricity and carbon film transfer for a-C:H(36) but not for a-C:H(20). Moreover, this range of *C*_H_ values is in line with the results of other studies that indicate that an effective passivation of self-mated a-C:H interfaces in UHV requires a rather high *C*_H_ ≈ 40 at. %.^19,20^ While a more systematic set of friction measurements considering more values of *C*_H_ would be necessary to determine more precisely the critical *C*_H_ above which superlubricity can be achieved, these measurements fall out of the scope of the present investigation, which mainly focuses on the mechanism of transfer film formation. Nonetheless, our results are in line with friction coefficients measured in previous studies for Si_3_N_4_ sliding against a-C:H with different *C*_H_. The comparison is shown in Figure 1c (details of the measurements are given in Table S1) and suggests a clear high-to-superlow friction transition for 20 < *C*_H_ < 35 at. %. A similar transition was also observed in the steel/a-C:H system in UHV.^16^ From a practical point of view, however, *C*_H_ values should be kept as low as possible, i.e., close to 30 at. %, because increasing *C*_H_ leads to lower elastic moduli and to polymeric-like structure^18^ of the a-C:H coatings, likely affecting their mechanical properties and lifetime. The introduction of hydrogen gradients in the a-C:H coating, i.e., higher hydrogen contents in topmost layers and lower hydrogen contents in the a-C:H bulk, would be ideal for controlling the thickness of a transfer film without renouncing a-C:H’s excellent mechanical properties.

## Conclusions

This study demonstrates the potential generality of the superlubricity mechanism which exploits the transfer of a carbon film onto a non-carbon countersurface and the superlubricious properties of a-C:H/a-C:H interfaces in UHV. Materials, such as silicon-based ceramics, which are not as tribologically performant as a-C:H coatings in a vacuum or dry conditions, can in principle be “protected” by a nanometer-thin carbonaceous transfer film, which ultimately can form a superlubricious sliding contact with the a-C:H-coated countersurface. A similar situation, in which superlubricity of Si_3_N_4_/Si_3_N_4_ systems was obtained in boundary lubrication with glycerol, was reported recently.^7^ In this case, both surfaces were protected by a nanometer-thin, N-containing carbon film which formed by tribochemical decomposition of the glycerol molecules. In the present case instead, the molecular source of carbon is replaced by a a-C:H-coated countersurface, which acts as a solid lubricant by enabling the transfer of an a-C:H nanofilm on the Si_3_N_4_ surface or SiO_2_ surface. A similar film-transfer process might be the reason why in several technological applications good tribological performances are obtained with heterogeneous interfaces in which only one of the two surfaces is coated with a DLC film.^36^ This offers obvious economic advantages, with respect to systems in which both surfaces are coated. Finally, we note that experimental results, in which superlubricity is detected also for SiC/a-C:H(36) and steel/a-C:H(36) systems (Figure S3), indicate the possible validity of this mechanism for other tribological interfaces. A generalization of our findings can make it possible to theoretically design other heterogeneous interfaces and atomically control the material transfer process and self-formation of superlubricious interfaces under unlubricated conditions. As discussed above, one of the main parameters to control the film transfer process is a-C:H’s hydrogen content. Potentially, introducing gradients of hydrogen content in the direction normal to the surface can also be exploited to introduce weak planes and facilitate film transfer. Further parameters might be a-C:H’s initial surface roughness and elastic modulus, as they ultimately control the contact area and the local pressure at contacts between surface asperities.^34^

## Materials and Methods

### Materials

Glass and Si_3_N_4_ balls are provided by CIMAP (Paris, France) and MetalBall (Grisolles, France). Glass balls are composed of 75% SiO_2_ and have a hardness of ∼4.4 GPa, an elastic modulus of 71 GPa, and a Poisson’s ratio of 0.17. Si_3_N_4_ balls are produced by hot pressing, where metal oxide binders are used. Even after sputtering, the surfaces are partially covered with a thin oxide layer and not a perfect crystal. We assume that initial contacts can occur between both the SiO_2_/a-C:H and Si_3_N_4_/a-C:H interfaces. The Si_3_N_4_ balls have a hardness of 1400–1600 HV, an elastic modulus of 310 GPa, and a Poisson’s ratio of 0.27. The diameters of the SiO_2_ and Si_3_N_4_ balls are 10 and 12 mm, respectively. a-C:H coatings with hydrogen content *C*_H_ = 20 and 36 at. %, called “a-C:H(20)” and “a-C:H(36)”, are coated on M2 steel substrates (with an elastic modulus of 207 GPa, a Poisson ratio of 0.29, and a Rockwell hardness of 64) by plasma-enhanced chemical vapor deposition. a-C:H(20) has a hardness of 27 GPa and an elastic modulus of 259 GPa,^34^ whereas a-C:H(36) has a hardness of 9 GPa and an elastic modulus of 80 GPa.^23^ The hydrogen contents of the a-C:H(20) and a-C:H(36) coatings are measured using Rutherford backscattering spectroscopy combined with forward recoil elastic scattering. The root-mean-square (RMS) roughness of the Si_3_N_4_, SiO_2_, a-C:H(20), and a-C:H(36) surfaces are measured using atomic force microscopy. The values of RMS roughness before and after sliding are given in Table S2.

### Tribological Tests and Surface Analyses

Both balls and flats are sonicated in heptane for 30 min and then in acetone for 5 min before introducing them into an UHV chamber. The Si_3_N_4_ and SiO_2_ balls are then sputtered by an Ar^+^ ion beam with 3 keV acceleration voltage in order to remove adventitious carbon. The sputtered area is 1 × 1 mm^2^. The survey spectrum is recorded every minute by XPS during the sputtering process, where a ULVAC-PHI Versa Probe II spectrometer conducts XPS. After sample preparation, ceramics and flats are installed in a controlled environment analytical tribology platform.^37^ This specific device is equipped with a tribometer with a new 6-axes force/torque sensor providing a unique opportunity to measure the unavoidable misalignment between the force sensor’s axes and that of the actuation used to load the contact. Only similar debiased signals can be safely used to characterize the superlow friction interfaces, like ceramics/DLC contacts under ultrahigh vacuum.^38^ This tribometer operates in a UHV chamber (where the residual gas pressure is 5 × 10^–7^ Pa) to avoid air contamination and ambient temperature. To ensure a maximum Hertzian contact pressure *P*_max_ of about 570 MPa, the normal force is applied as 1.9 N for Si_3_N_4_/a-C:H and 7.7 N for SiO_2_/a-C:H. The diameters of initial Hertzian circular contacts are about 160 and 80 μm for SiO_2_ and Si_3_N_4_, respectively. The average sliding speed is 0.2 mm s^–1^ (1° s^–1^ along 20°) for both systems with rotational reciprocating sliding.

After tribotests, samples are directly transferred to the XPS/XAES chamber in UHV. XPS/XAES spectra of samples are collected by analyzing a 50 × 50 μm^2^ area at a takeoff angle of 45° and a pass energy of 23.5 eV. Subsequently, the samples are retrieved from UHV and imaged by digital microscopy (VHX-6000, Keyence, Osaka, Japan). Raman spectra (Invia Raman spectroscopy, Renishaw, UK) are recorded on those samples except for the Si_3_N_4_ ball because no visible traces exist on the Si_3_N_4_ ball. A 633 nm laser is adopted during spectrum accumulation with a power of less than 10 mW to avoid a laser empowered structure change.

### Quantum-Mechanical Molecular Dynamics Simulations

Two types of MD simulations are performed by using the self-consistent-charge density-functional tight-binding (SCC-DFTB) method^30^ as implemented in the Atomistica package (https://github.com/Atomistica). The first type of simulation is bulk shearing of a-C:H (Figure 4), and the other type is sliding at Si_3_N_4_/a-C:H and a-SiO_2_/a-C:H interfaces (Figures 5 and 6). SCC-DFTB is an approximate method to density-functional theory, which allows a drastic reduction of the computational effort while retaining a level of accuracy that is sufficient to describe chemical processes in various materials systems, including those presented in this work. An introduction to this method suitable for a broad audience is provided in ref (39). Here, we use the Slater–Koster parameters pbc-0-3^40,41^ that are well tested especially for Si_3_N_4_ surfaces,^7,41^ silica/diamond,^42^ and ta-C/glycerol/ta-C tribological interfaces.^43^ The ground-state Schrödinger equations are explicitly solved to obtain the potential energy, and the forces acting on each atom are evaluated from the first derivatives of the energy with respect to nuclear coordinates according to the Hellmann–Feynman theorem.^44^ Using these forces, the motion of atomic nuclei is described using Newton’s equation of motion. This quantum-chemical MD approach is different from classical MD with reactive interatomic potentials and is computationally far more expensive. However, classical MD cannot reliably describe the complex interfacial phenomena involving bond formation and breaking such as the tribochemical processes^45^ investigated in this work, and therefore quantum-chemical methods are state-of-the-art techniques to model such systems.^46,47^

Bulk a-C:H samples with densities between 1.6 and 2.1 g cm^–3^ are prepared by rapidly quenching melts from 5000 to 0 K at a constant rate (*Ṫ* = 10^14^ K s^–1^) while keeping the system volume constant using the SCC-DFTB method. The densities decrease linearly from 2.1 to 1.6 g cm^–3^ as *C*_H_ increases.^18^ Different lateral dimensions are employed to simulate two interfaces: 10.0 × 10.0 Å^2^ for a-SiO_2_/a-C:H and 15.6 × 13.5 Å^2^ for Si_3_N_4_/a-C:H. a-SiO_2_ bulk models with a density of about 2.5 g cm^–3^ and lateral dimensions of 10.0 × 10.0 Å^2^ (consisting of 40 Si and 80 O atoms) are prepared following the quench-from-the-melt protocol described in ref (48) using the interatomic potential by Vashishta et al.^49^ as implemented in LAMMPS.^50^ The β-Si_3_N_4_ (0001) slab has a lateral dimension of 15.6 × 13.5 Å^2^ and consists of 72 Si and 96 N atoms. To simulate the Si_3_N_4_/a-C:H and a-SiO_2_/a-C:H sliding interfaces, the slabs are obtained by releasing the periodic BCs along the *z* direction. The dangling bonds of the atoms in the outermost layers far from the sliding interface are terminated with hydrogen atoms or a hydroxyl group (only for Si of a-SiO_2_), while those at the interface are not passivated.

For the 0.1 ns a-C:H simulations (Figure 4) to determine the yield stress for plastic shear flow of representative bulk volumes under uniform shear deformation, we use Lees–Edwards BCs.^31^ A schematic image of the Lees–Edwards BCs is provided in Figure 4a. This simulation model represents high-friction, cold-welded interfaces between surface asperities during running-in. A Berendsen barostat^32^ and a Peters thermostat^33^ are employed to keep the system at a constant pressure of 5 GPa and at a constant temperature of 300 K, respectively. The yield shear stress to generate plastic shear flow is calculated as the shear stress of bulk materials and is different from the shear stress of the same materials after the formation of localized, low-friction interfaces.^23^ Bulk a-C:H can form low-friction interfaces under shear, as shown in Figure 6. However, such shear localization leading to a drastic drop in the shear stress is not considered in Figure 4, where yield shear stresses are calculated before the formation of any low-friction interface. To calculate the yield shear stress of Si_3_N_4_ and SiO_2_, we employ the BCs shown in Figure 5a. These simulations are performed at 5 GPa and 300 K. We checked that the calculated shear stresses are not affected by the choice of BCs for a-C:H.

For sliding simulations of Si_3_N_4_/a-C:H and a-SiO_2_/a-C:H interfaces (see an example in Figure 5a), we keep the two outermost layers and the terminating hydrogen atoms of the lower slab fixed, while a rigid layer of the upper slab with the same thickness is driven at a constant velocity of 100 m s^–1^ after an initial temperature and normal load equilibration. The normal pressure *P*_*z*_ is applied to the topmost rigid layer using a pressure-coupling algorithm developed by Pastewka et al.^35^ A higher temperature of 500 K is employed with a Langevin thermostat^51^ on all nonrigid atoms. The thermostat is applied only along the direction perpendicular to the sliding direction and to normal pressure *P*_*z*_. Contact pressures *P*_*z*_ of 1 and 5 GPa are used. After 0.5 ns of sliding at the constant pressures, we simultaneously remove the normal pressures and add a constant vertical speed of 20 m s^–1^ to the topmost rigid layer while keeping the sliding speed constant to simulate transfer film formation. The equations of motion are integrated with a time step Δ*t* = 0.5 fs using the velocity-Verlet algorithm.^51^ Five independent MD trajectories are generated for each set of parameters. The length and time scales of the SCC-DFTB MD simulations are orders of magnitude smaller than those of the UHV experiment, and thus, it is not possible to quantitatively compare the friction coefficient recorded experimentally with that obtained from the MD simulations. However, the atomistic simulations provide an understanding of the tribochemical mechanisms underlying the formation of a carbonaceous transfer film occurring at the scale of single-asperity contacts and qualitative agreement with the macroscopic experiments regarding the dependency of the transfer film formation on the a-C:H’s hydrogen content. Because the local contact pressure at the contact between surface asperities is significantly larger than the Hertzian contact pressure,^34^ in the atomistic simulations we use local normal pressure values of the order of 1 GPa, which is a typical value at this scale according to estimates based on AFM surface topographies and contact mechanics simulations.^7,34^

## Supporting Information Available

Figure S1: chemical compositions of the Si_3_N_4_ and SiO_2_ balls as a function of sputtering time using XPS; Figure S2: reciprocating friction tests of Si_3_N_4_/a-C:H(36) at different normal loads; Figure S3: reciprocating friction tests of SiC/a-C:H(36) and steel/a-C:H(36); Figure S4: Raman spectra measured after sliding of the a-C:H(36)-coated steel flat against the SiO_2_ ball; Figure S5: XPS N1s recorded before sliding on the sputtered Si_3_N_4_ ball and inside the wear scar on the Si_3_N_4_ ball; Figure S6: time evolution of the shear stress τ during 0.5 ns SCC-DFTB MD sliding simulations; Figure S7: example of atomic configurations of the SiO_2_/a-C:H interface after sliding at 1 GPa and subsequent detachment; Figure S8: evolution of chemical structures and velocity profiles for the SiO_2_/a-C:H interface at a contact pressure of 1 GPa; Table S1: a summary of experimental details of previous studies shown in Figure 1c; Table S2: root-mean-square (RMS) roughness of the Si_3_N_4_, SiO_2_, a-C:H(20), and a-C:H(36) surfaces measured before and after sliding using atomic force microscopy (PDF)
