# Supplementary material for: Superlubricity of Silicon-Based Ceramics Sliding against Hydrogenated Amorphous Carbon in Ultrahigh Vacuum: Mechanisms of Transfer Film Formation
Source: ACS Appl Mater Interfaces. 2024 Jan 31;16(6):8032–44. doi: 10.1021/acsami.3c16286 (PMC10876050; doi:10.1021/acsami.3c16286)
Supplement: Supplementary file 1 — am3c16286_si_001.pdf [file am3c16286_si_001.pdf]

Supporting Information

Superlubricity of Silicon-Based Ceramics Sliding against Hydrogenated Amorphous Carbon in Ultrahigh Vacuum: Mechanisms of Transfer Film Formation

Takuya Kuwahara[1,2], Yun Long[3], Aslihan Sayilan[3], Thomas Reichenbach[1], Jean Michel Martin[3], Maria-Isabel De Barros Bouchet[3], Michael Moseler[1,4,5,6*], and Gianpietro Moras[1]

[1]*Fraunhofer IWM, MikroTribologie Centrum µTC, Wöhlerstraße 11, 79108 Freiburg, Germany*

[2]*Department of Mechanical Engineering, Osaka Metropolitan University, 3-3-138 Sugimoto, Sumiyoshi-ku, 558-8585 Osaka, Japan*

[3]*Laboratory of Tribology and System Dynamics, Ecole Centrale de Lyon, 69134 Ecully, France*

[4]*Institute of Physics, University of Freiburg, Hermann-Herder-Straße 3, 79104 Freiburg, Germany*

[5]*Freiburg Materials Research Center, University of Freiburg, Stefan-Meier-Straße 21, 79104 Freiburg, Germany*

[6]*Cluster of Excellence livMatS, Freiburg Center for Interactive Materials and Bioinspired Technologies, University of Freiburg, Georges-Köhler-Allee 105, 79110 Freiburg, Germany*

[*]Corresponding author: michael.moseler@iwm.fraunhofer.de

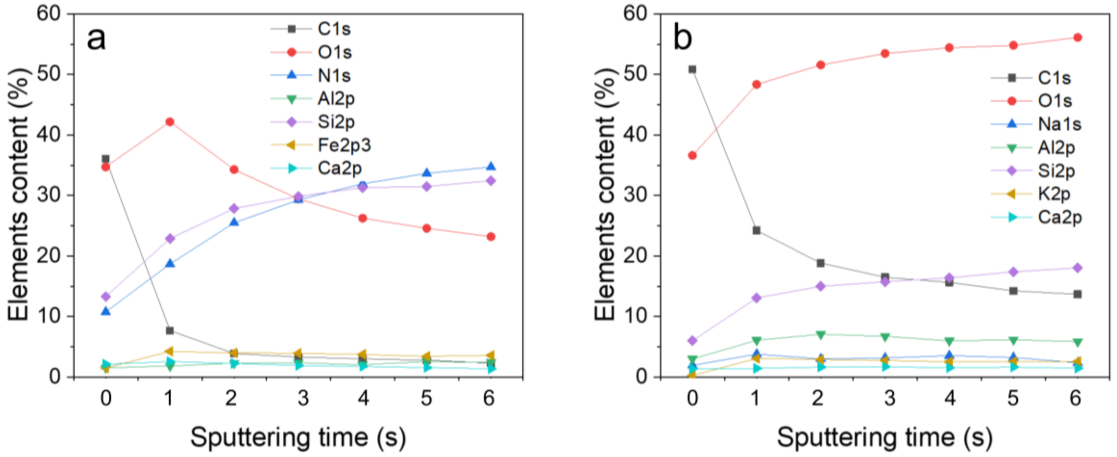

Figure S1. Chemical compositions of (a) the Si_3N_4 and (b) SiO_2 ball as a function of sputtering time using XPS. The details are described in Methods.

Table S1. A summary of experimental details of previous studies shown in Fig. 1c.

Label	H content (at. %)	Environments	Friction coefficient	Parameters
Li 2007[1]	0	Dry N_2 (RH 5%)	> 0.5	2 N, 125 m/min
Cui 2013[2]	8	High vacuum (2×10^{-4} Pa)	0.6	1 N, 200 rpm
This study	20	UHV (5×10^{-9} Pa)	0.84	1.9 N, 0.2 mm/s
Xia 2008[3]	26.7	High vacuum (2×10^{-4} Pa)	0.1	2 N, 40 rpm
This study	36	UHV (10^{-9} Pa)	0.008±0.002	1.9 N, 0.2 mm/s
Li 2007[1]	39[4]	Dry N_2 (RH 5%)	0.01	2 N, 125 m/min
Chen 2017[5]	39.3	Dry N_2	0.008	2 N (0.68 GPa), 0.15 m/s

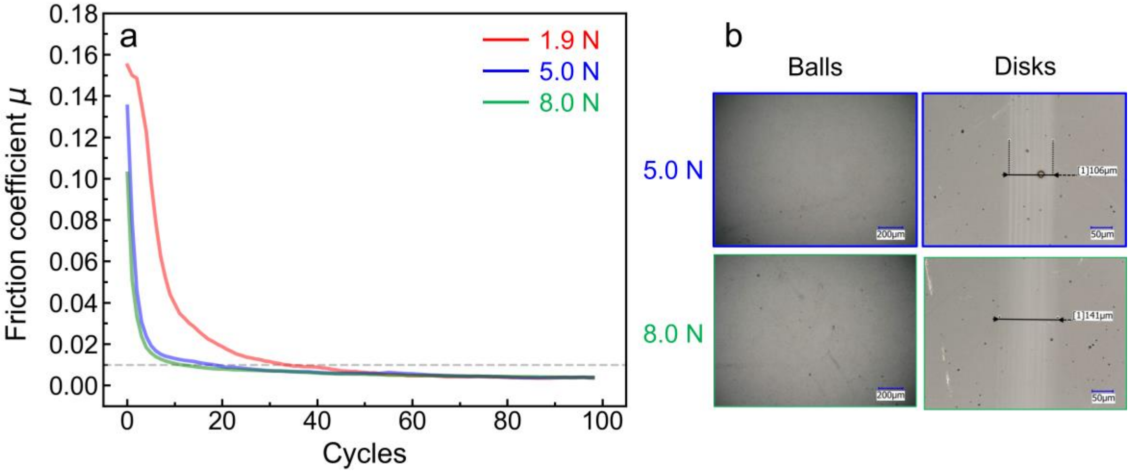

Figure S2. Reciprocating friction tests of Si_3N_4/a-C:H(36) at different normal loads: 1.9 (red), 5.0 (blue), and 8.0 (green) N. The corresponding maximum Hertzian contact pressures are 571, 788, and 921 MPa, respectively. All friction tests are conducted in the UHV chamber (where the residual gas pressure is 5×10^{-7} Pa). (a) Friction curves and (b) optical images of the wear scars. The red curve for 1.9 N is one of the three curves shown in Fig. 1b.

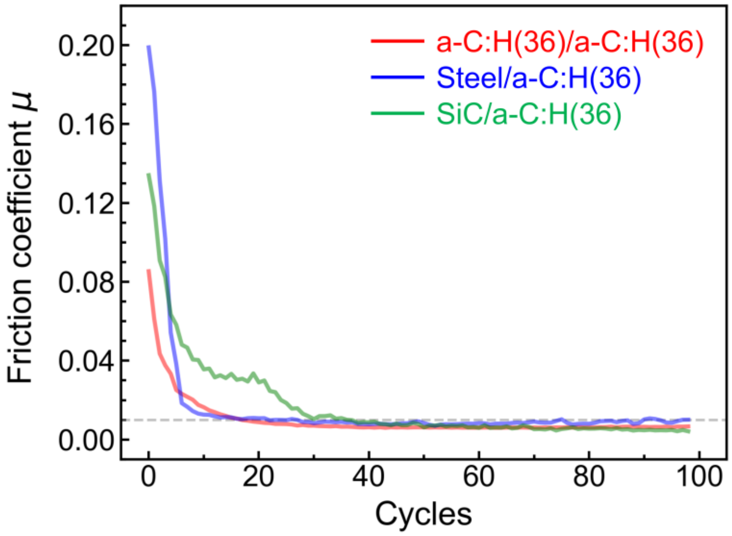

Figure S3. Reciprocating friction tests of SiC/a-C:H(36) (green) and steel/a-C:H(36) (blue) as well as self-mated a-C:H(36) (red). The maximum Hertzian contact pressure of about 570 MPa are identical for all three systems. The SiC ball with a diameter of 11.1 mm and steel ball with a diameter of 12.7 mm are provided by Total (Paris, France) and MetalBall (Grisolles, France), respectively. The SiC ball has an elastic modulus of 410 and Poisson ration of 0.18. The steel ball has an elastic modulus of 210 GPa and Poisson ratio of 0.3.

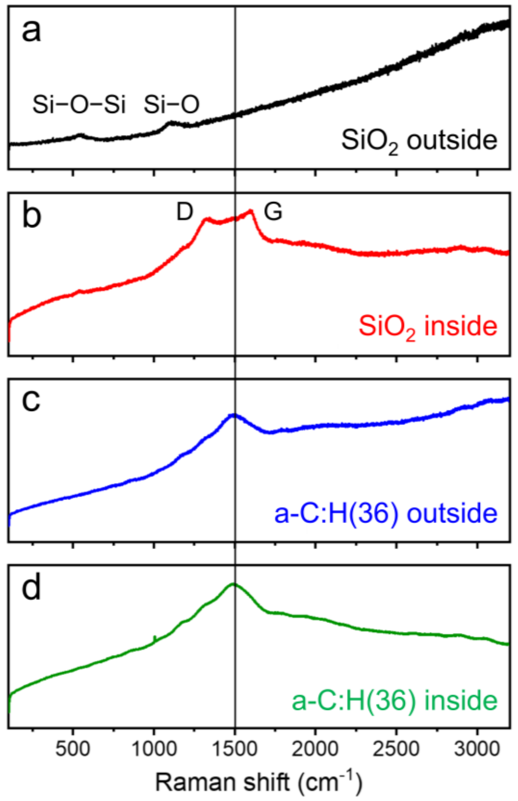

Figure S4. Raman spectra measured after sliding of the a-C:H(36)-coated steel flat against the SiO_2 ball: (a) outside and (b) inside the wear scar on the SiO_2 ball, and (c) outside and (d) inside the wear scar on the a-C:H(36)-coated steel disc.

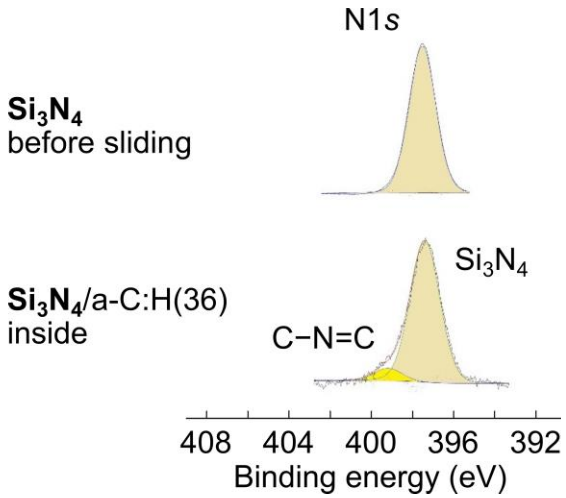

Figure S5. XPS N1*s* recorded before sliding on the sputtered Si_3N_4 ball (top) and inside the wear scar on the Si_3N_4 ball (bottom). The peak position corresponding to Si_3N_4 is located at 397.5 eV.

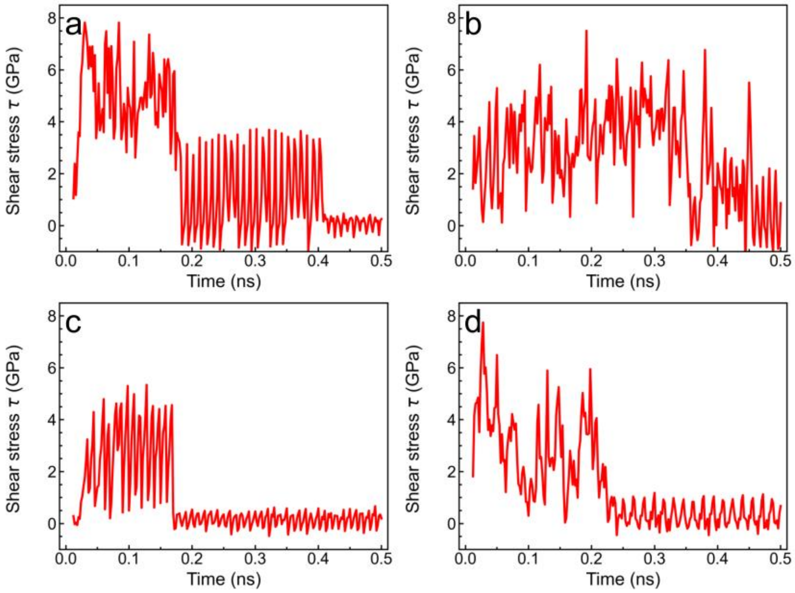

Figure S6. Evolution of the shear stress τ averaged every 2 ps for 0.5-ns sliding. The trajectories in panel a−d are identical with those in Fig. 6a−d, respectively.

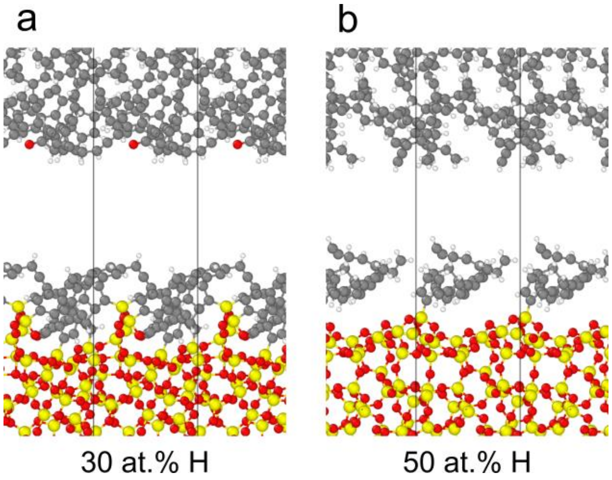

Figure S7. Examples of atomic configurations of the SiO_2/a-C:H interface after sliding at 1 GPa and subsequent detachment. Panel a and b correspond to the systems in Fig. 6a and c, respectively.

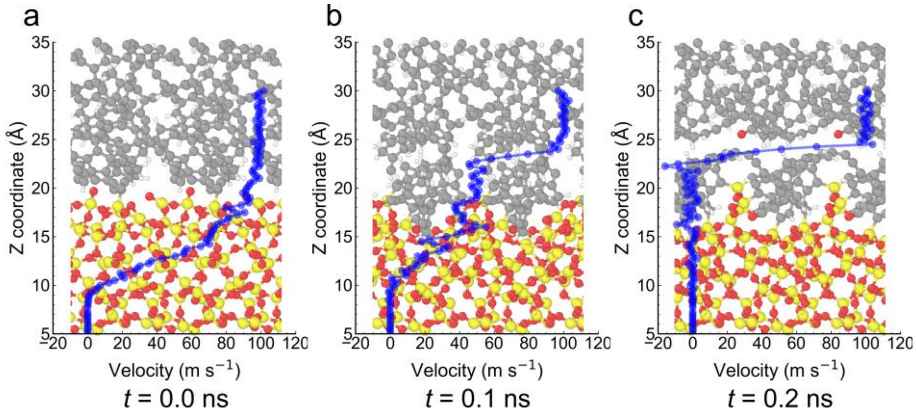

Figure S8. Evolution of chemical structures and velocity profiles for the SiO_2/a-C:H interface at a contact pressure of 1 GPa: (a) t = 0.0, (b) 0.1, and (c) 0.2 ns. This system is identical to the one shown in Figs. 6a and S7a.

Table S2. Root-mean-square (RMS) roughness of the Si_3N_4, SiO_2, a-C:H(20), and a-C:H(36) surfaces measured before and after sliding using atomic force microscopy. Lower friction coefficients of the Si_3N_4/a-C:H(36) than the SiO_2/a-C:H(36) (Fig. 1b) can be due to the smaller RMS roughness of the Si_3N_4 surface than the SiO_2 surface.

Before sliding		
Materials	RMS roughness (nm)	
Si_3N_4	9.1	
SiO_2	44.0	
a-C:H(20)	8.8	
a-C:H(36)	14.3	
After sliding		
Systems	Materials	RMS roughness (nm)
Si_3N_4/a-C:H(20)	Si_3N_4	15.9
	a-C:H(20)	57.7
SiO_2/a-C:H(20)	SiO_2	45.1
	a-C:H(20)	8.2
Si_3N_4/a-C:H(36)	Si_3N_4	6.5
	a-C:H(36)	9.1
SiO_2/a-C:H(36)	SiO_2	40.0
	a-C:H(36)	12.2

References

(1) Li, H.; Xu, T.; Wang, C.; Chen, J.; Zhou, H.; Liu, H. Tribochemical Effects on the Friction and Wear Behaviors of a-C:H and a-C Films in Different Environment. *Tribol. Int.* **2007**, *40* (1), 132–138. https://doi.org/10.1016/j.triboint.2006.03.007.

(2) Cui, L.; Lu, Z.; Wang, L. Toward Low Friction in High Vacuum for Hydrogenated Diamondlike Carbon by Tailoring Sliding Interface. *ACS Appl. Mater. Interfaces* **2013**, *5* (13), 5889–5893. https://doi.org/10.1021/am401192u.

(3) Xia, L.; Li, G. The Frictional Behavior of DLC Films against Bearing Steel Balls and Si_3N_4 Balls in Different Humid Air and Vacuum Environments. *Wear* **2008**, *264* (11–12), 1077–1084. https://doi.org/10.1016/j.wear.2007.08.010.

(4) Johnson, J. A.; Woodford, J. B.; Chen, X.; Andersson, J.; Erdemir, A.; Fenske, G. R. Insights into "Near-Frictionless Carbon Films". *J. Appl. Phys.* **2004**, *95* (12), 7765–7771. https://doi.org/10.1063/1.1739287.

(5) Chen, X.; Zhang, C.; Kato, T.; Yang, X.; Wu, S.; Wang, R.; Nosaka, M.; Luo, J. Evolution of Tribo-Induced Interfacial Nanostructures Governing Superlubricity in a-C:H and a-C:H:Si Films. *Nat. Commun.* **2017**, *8*, 1675. https://doi.org/10.1038/s41467-017-01717-8.
